# Comparison of Skull Motions in Six Degrees of Freedom Between Two Head Supports During Frameless Radiosurgery by CyberKnife

**DOI:** 10.3389/fonc.2018.00359

**Published:** 2018-09-04

**Authors:** Chen-Lin Kang, Shyh-Chang Liu, Jui-Chu Wang, Kuan-Cho Liao, Yu-Jie Huang, Fu-Min Fang, Tsung-I Liao, Kuo-Jung Juan, Chun-Chieh Huang

**Affiliations:** ^1^Department of Radiation Oncology, Kaohsiung Chang Gung Memorial Hospital, Chang Gung University College of Medicine, Kaohsiung, Taiwan; ^2^Department of Information Engineering, I-Shou University, Kaohsiung, Taiwan; ^3^Department of Anatomical Pathology, Kaohsiung Chang Gung Memorial Hospital, Chang Gung University College of Medicine, Kaohsiung, Taiwan; ^4^Graduate Institute of Clinical Medical Sciences, College of Medicine, Chang Gung University, Taoyuan, Taiwan

**Keywords:** CyberKnife, frameless, stereotactic radiosurgery, immobilization, intrafractional motion

## Abstract

**Introduction:** Maintaining immobilization to minimize skull motion is important during frameless radiosurgery. This study aimed to compare the intrafractional skull motions between two head supports.

**Methods:** With 6D skull tracking system, 4,075 image records from 45 patients receiving radiosurgery by CyberKnife were obtained. Twenty-three patients used TIMO head supports (CIVCO) (Group A) and twenty-two patients used Silverman head supports (CIVCO) with MoldCare cushions (ALCARE) (Group B). The skull motions in X (superior-inferior), Y (right-left), Z (anterior-posterior) axes, 3D (three-dimensional) vector, Roll, Pitch and Yaw between the two groups were compared and the margins of planning target volume were estimated.

**Results:** The translational motions in Group A were similar in three axes at initial but became different after 10 min, and those in Group B were less prominent in the Y axis. The rotational errors in Group A were most obvious in Yaw, but those in Group B were stationary in three axes. The motions in the X axis, 3D vector, Pitch and Yaw in Group B were significantly smaller than those in Group A; conversely, the motions in the Z axis in Group B were larger. To cover the 95% confidence intervals, margins of 0.77, 0.79, and 0.40 mm in the X, Y, and Z axes, respectively, were needed in Group A, and 0.69, 0.50, and 0.51 mm were needed in Group B.

**Conclusions:** Both head supports could provide good immobilization during the frameless radiosurgery. Silverman head support with MoldCare cushion was better than TIMO head support in the superior-inferior direction, 3D vector, Pitch and Yaw axes, but worse in the anterior-posterior direction.

## Introduction

The CyberKnife robotic radiosurgery system is one of the frameless image-guided radiosurgery techniques (1–3). Patients do not need fixation with uncomfortable frames, and the overall accuracy of frameless image-guided radiosurgery is similar to that of conventional frame-based radiosurgery (4).

To achieve high accuracy in frameless radiosurgery, good immobilization during the entire course is very important. Unlike frame-based radiosurgery, frameless radiosurgery uses a thermoplastic mask with a suitable head support for immobilization, which does not eliminate intrafractional errors (5–7).

There are two kinds of head supports in our department for application in frameless radiosurgery. One kind of head support is the standard pillow without a cushion, and the other is a pillow with a cushion. However, the optimal head support for immobilization during the frameless radiosurgery is unknown. The choice of head support for each patient is dependent on the physician's preference.

Consequently, we retrospectively collected the data of alignment and image-guidance from patients undergoing frameless radiosurgery by CyberKnife to compare the skull motion in six degrees of freedom during the treatment between two head supports. In addition, we further proposed margins for the planning target volume (PTV), and assessed whether each margin was sufficient to cover the intrafractional error in each axis.

## Materials and methods

### Patients and data collection

This study was approved by the institutional review board in our institution (No. 201601207B0). The need for informed consent from each patient was waived by the institutional review board, because this study was non-invasive and utilized routine treatment data.

Forty-five patients who received intracranial radiosurgery during the period from July 2015 to August 2016 were enrolled. The diagnoses of these patients were as follows: 22 metastatic brain tumors, 3 meningiomas, 4 arteriovenous malformations, 10 arteriovenous fistulas, 2 acoustic neuromas, 1 pituitary tumor, and 3 carotid-cavernous fistulas. The doses were prescribed to 80–90% isodose line and the prescription doses ranged from 12 to 44 Gy in 1 to 5 fractions, with a mean dose of 20.0 Gy.

We obtained the image data for these patients from the CyberKnife Data Management System. The data for 33 patients were fully acquired due to the skull motions within the manipulator-correctable range throughout the treatment. The manipulator of CyberKnife M6 system could correct translations of up to 10 mm in the 3 translational axes and rotations of up to 1.0° in Roll and Pitch and 3.0° in Yaw (8, 9) The data of the other 12 patients were acquired from the beginning of treatment to the first correction by moving the couch due to the skull motion exceeding the upper limit of manipulator-correctable range. Consequently, we could ensure that all the data had the same baseline without moving the couch. The treatment time ranged from 23 to 86 min, with an average time of 48.4 min. The mean interval of each image taken was 32.1 s. Finally, a total of 4075 images were obtained.

### Head supports for immobilization

**Figure 1:**
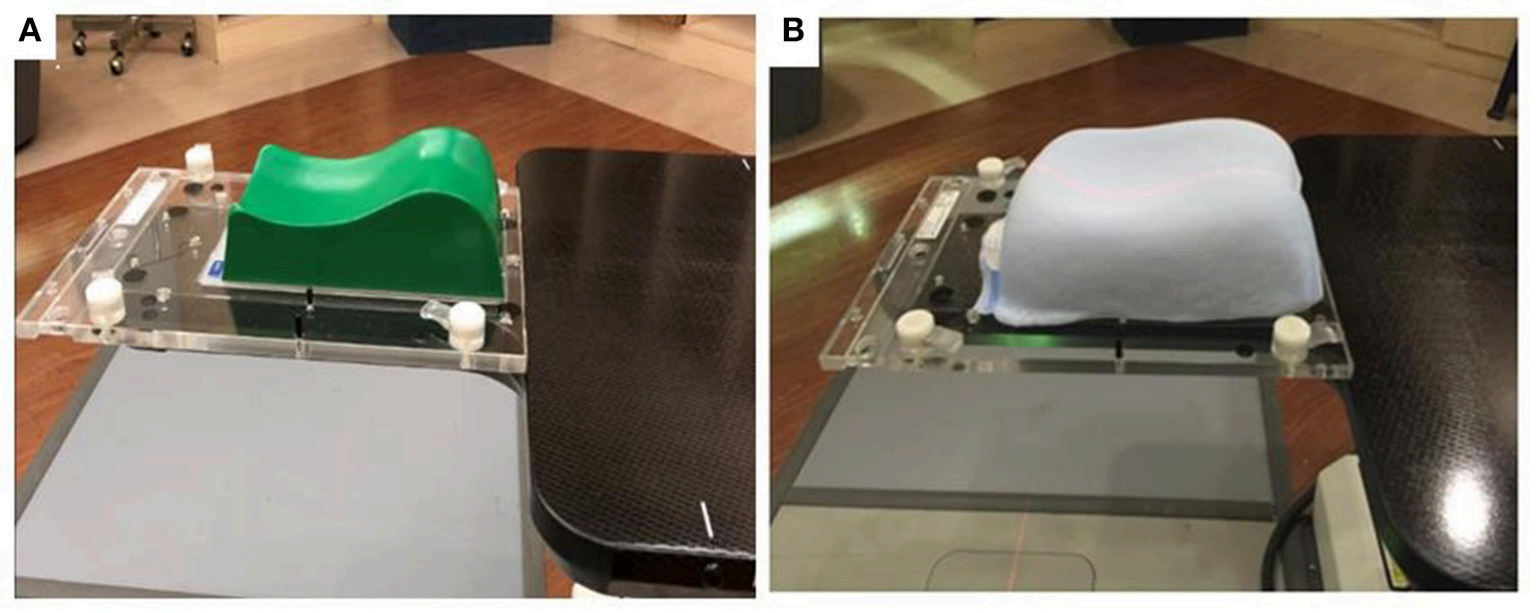
**(A)** TIMO head support in the group A. (**B)** Silverman head support with MoldCare cushion in the group B.

Patients were immobilized in the supine and head-first position, with a 2.4-mm-thick U-Frame thermoplastic mask. There were two kinds of head supports in our department, and the kind of head support use was dependent on the physician's preference. Twenty-three patients (Group A) used TIMO head supports (CIVCO Medical Solutions, Coralville, IA, USA), which were made of molded polyurethane foam with a washable coating to provide head and neck support. The other twenty-two patients (Group B) used Silverman head supports (CIVCO Medical Solutions, Coralville, IA, USA) with MoldCare cushions (ALCARE Co., Tokyo, Japan) (Figure 1). The patient characteristics in these two groups were shown as Table 1. The Silverman head support was made of thin clear plastic material and the MoldCare cushion was a soft fabric bag containing polystyrene beads coated with moisture-cured polyurethane resin. When sprayed with room temperature water, the cushion became moldable. Five-to-ten minutes later, the cushion hardened to form a rigid custom support. The computed tomography (CT) scan was carried out with 125 kVp, 400 mA and thickness of 1.25 mm per slice for the planning.

**Table 1:** Patient characteristics.

**Characteristics**	**Group A**	**Group B**	***P*-value**
	***n* = 23, *n* (%)**	***n* = 22, *n* (%)**	
Age (Mean, *SD*)	61.4 (11.8)	58.0 (15.6)	0.402^a^
Gender			
Male	12 (52.2)	14 (63.6)	0.436^b^
Female	11 (47.8)	8 (36.4)	
Collimator			0.999^c^
InCise MLC	14 (60.9)	15 (68.2)	
Iris variable aperture	8 (34.8)	7 (31.8)	
Fixed cone	1 (4.3)	0 (0.0)	
Treatment time (Mins) (Mean, *SD*)	45.3 (12.8)	51.7 (15.5)	0.137^a^
Diagnosis			0.128^c^
Acoustic neuroma	1 (4.3)	1 (4.5)	
Arteriovenous fistula	3 (13.0)	7 (31.9)	
Arteriovenous malformation	2 (8.7)	2 (9.1)	
Carotid cavernous fistula	2 (8.7)	1 (4.5)	
Meningioma	0 (0.0)	3 (13.6)	
Metastatic brain tumor	15 (65.3)	7 (31.9)	
Pituitary tumor	0 (0.0)	1 (4.5)	

SD, standard deviation; MLC, Multileaf Collimator; Mins, minutes.

a The P value calculated from Independent T-test.

b The P value calculated from Chi-square test.

c *The P value calculated from Fisher exact test*.

### Robotic radiosurgery system

The CyberKnife M6 system (Accuray, Inc., Sunnyvale, CA, USA) consists of an X-band cavity magnetron and a side-coupled standing wave linear accelerator mounted on a robotic manipulator. The linear accelerator produces an unflattened 6 MV photon beam with a dose rate up to 1000 cGy/min. The beam can be collimated by either one of 12 fixed circular tungsten cones with diameters ranging from 5 to 60 mm or Iris variable aperture collimator with the same set of field sizes as fixed cones or an InCise multi-leaf collimator. The M6 series is the first CyberKnife system to have the multi-leaf collimator, which could reduce the total treatment time (8).

The image-guided system consists of two x-ray sources mounted in the ceiling and two amorphous silicon flat-panel detectors embedded in the floor, imaging the patient from two orthogonal views at ±45° oblique angles. The amorphous silicon flat-panel X-ray detectors generate a high-resolution digital image (1024 × 1024 pixels and pixel pitch 400 μm). The target localization during patient setup and treatment is achieved by comparing the live camera images with a series of digitally reconstructed radiographs (DRRs) generated from the planning CT at 45° angles through the imaging center. Based on this comparison, the tracking software calculates the differences in the three translational and three rotational axes between simulation and treatment positions.

### Axes definition and 6D skull tracking system

The coordinates of all axes are based on the supine position on the treatment table. Consequently, the X axis is the patient's superior-inferior (SI) direction, positive toward the feet and negative toward the head, the Y axis is the patient's right-left (RL) direction, positive toward the left and negative toward the right, and the Z axis is the patient's anterior-posterior (AP) direction, positive toward the abdomen and negative toward the back. The rotations are defined as follows: Roll is based on the X axis, turning right is positive and turning left is negative; Pitch is based on the Y axis, the head raising is positive and the foot raising is negative; and Yaw is based on the Z axis, rotating counterclockwise is positive and rotating clockwise is negative. Before treatment, the patient was aligned using an adjustable couch to reduce the corrections to below the maximum robotic manipulator limits.

**Figure 2:**
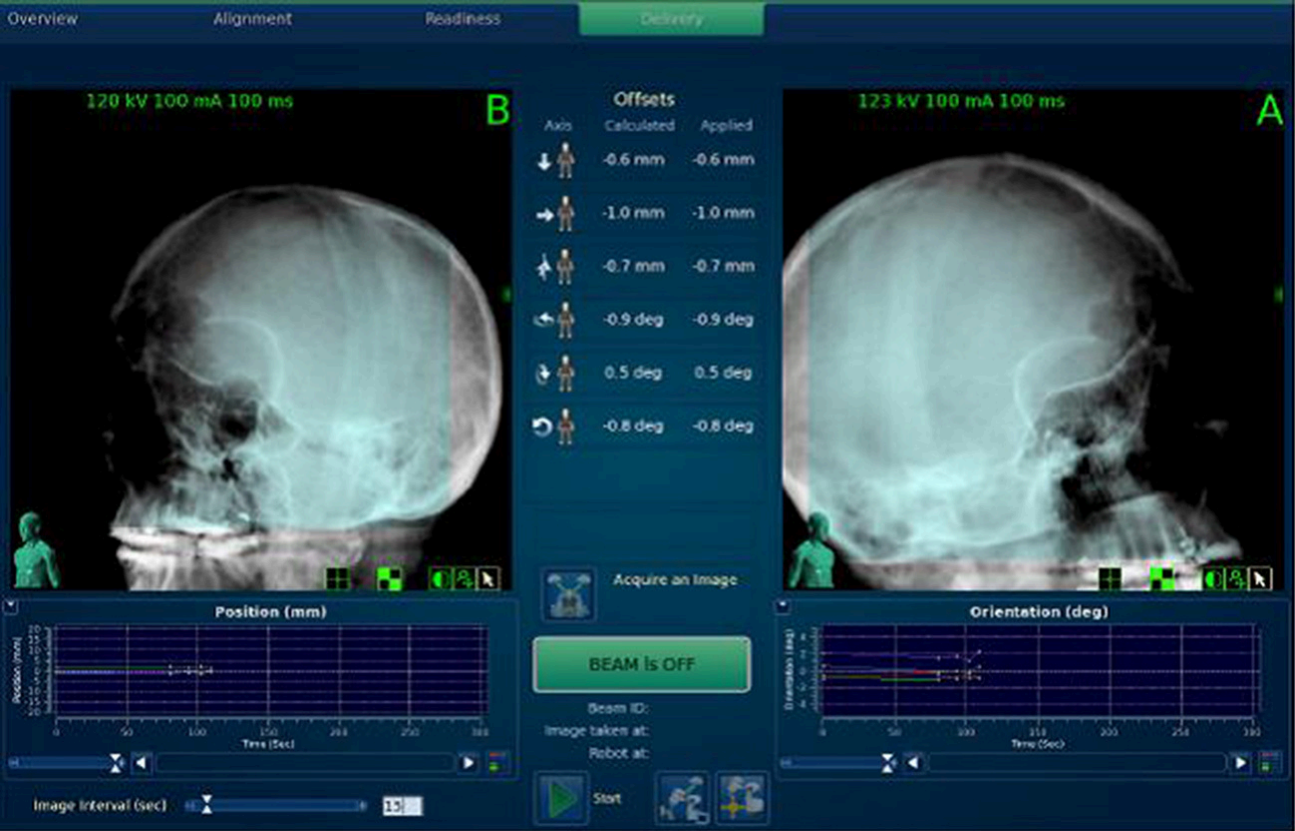
Using 6D skull tracking system to identify the skull and track the skull motion.

The 6D skull tracking mode was used to identify the skull and track the skull motion in six degrees of freedom based on the fixed relationship between the target volume and the skeletal features of the skull (Figure 2) (9). The image alignment must be performed to achieve a position error less than 1 mm and 1° before starting the treatment. The treatment location system compares sets of live camera images with a series of DRRs. During treatment, the system allows the users to specify the minimum time interval between each image acquisition within a range of 15–150 s during treatment. In our hospital, a default of 15 s intervals between each image acquisition is used when the treatment starts, while the interval can increase gradually up to 45 s after verifying the stability for the first few min.

### Data analysis and statistics

We set every 2 min as a time point and analyzed the data up to 40 min. The data of 3 images (previous one, this one and next one) around each time point were taken to reduce the influence from extreme values measured by a single image. To fully present the trends of intra-fractional skull motion, the deviation analysis took the data of the first image as baseline and calculated the deviation. The mean deviations of three images around each time point for each patient could be averaged to obtain the errors at each time point of six axes (X, Y, Z, Roll, Pitch, and Yaw). The absolute deviations in three translational axes at each time point for each patient were used to calculate the three-dimensional (3D) vector error as follows:

$$\begin{array}{l} 3\text{D vector}=\sqrt{SI^{2}+RL^{2}+AP^{2}} \end{array}$$

The progressive changes in errors by the treatment time between these two groups were compared by dividing the data of 40 min into four 10-min (10-min) time sessions. The Friedman ANOVA test was used to analyze the changes of errors across these 10-min time sessions. We used linear regression analysis to evaluate the difference between two groups. The statistical analyses were performed by IBM SPSS Statistics 22.0 (IBM Corp., Armonk, NY, USA). The data were used to calculate the 3D margin for clinical target volume (CTV) to become PTV according to the previous literature proposed by van Herk et al. (10) The PTV margin (Mptv) was estimated to ensure that 90% of the treatment plan volume was covered by 95% of the prescribed dose as follows:

$$\begin{array}{l} \text{Mptv}=2.5\Sigma +0.7\sigma \end{array}$$

The system error (Σ) was the standard deviation of the average of each treatment record, and the random error (σ) was the mean square root of each treatment record average.

Finally, the cumulative frequency of errors with continuous increments of 0.1 mm of deviation in the translational axes were plotted to evaluate both the 95% confidence intervals of skull motions and the coverage by PTV margin of 1 mm in clinical practice.

## Results

**Figure 3:**
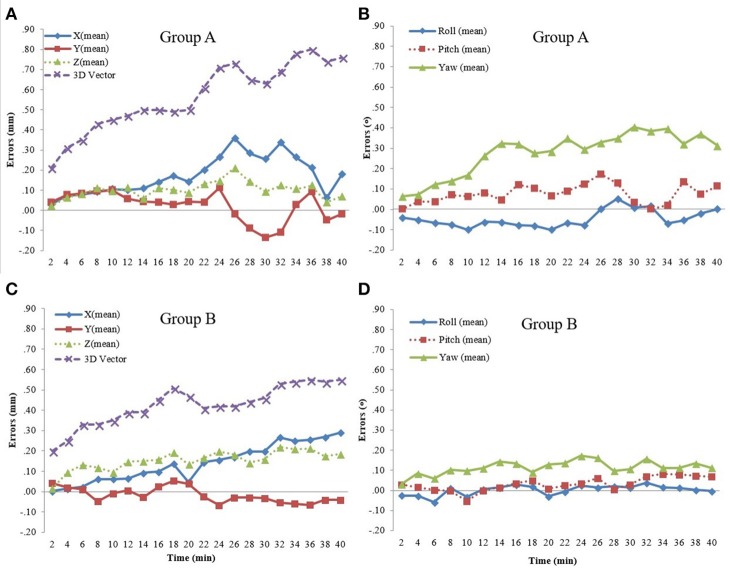
**(A)** Translational errors in Group A. **(B)** Rotational errors in Group A. **(C)** Translational errors in Group B. **(D)** Rotational errors in Group B.

With an interval of 2 min, the panoramic pictures of translational and rotational errors within 40 min in both groups are shown in Figure 3. In Group A, the translational errors in the three axes were similar within 10 min, but differences appeared thereafter. In Group B, the translational error in the Y axis was less prominent than that in the X and Z axes. On the other hand, the most obvious rotational error in Group A was in Yaw, while those in Group B were much smaller and more stable in the three axes during the treatment.

The progressive changes of translational and rotational errors divided into four 10-min sessions are shown in Table 2. The significant changes in translational errors in Group A were in the X axis (*P* = 0.045) and 3D vector (*P* < 0.001), while those in Group B were both in the X (*P* = 0.022) and Z axes (*P* < 0.001), and in the 3D vector (*P* < 0.001). As for rotational errors, there were no significant changes in either group. To compare between the groups, we found that the changes in errors in the X axis (*P* = 0.001), 3D vector (*P* = 0.014), Pitch (*P* = 0.041), and Yaw (*P* = 0.032) in Group B were significantly smaller than those in Group A. However, the change in translational error in the Z axis (*P* = 0.031) in Group B was significantly larger than that in Group A.

**Table 2:** Progressive changes of translational and rotational errors within 0–40 min in different head supports divided into four 10-min sessions.

**Axes**	**Group**	**Mean errors in 10-min sessions**				**P^a^**	**B^b^**	**P^c^**
		**0–10**	**10–20**	**20–30**	**30–40**			
X	A	0.06	0.12	0.20	0.26	0.045	−0.038	0.001
	B	0.03	0.08	0.15	0.23	0.022		
Y	A	0.06	0.09	0.02	−0.01	0.582	−0.025	0.307
	B	0.01	0.00	−0.03	−0.04	0.541		
Z	A	0.06	0.10	0.13	0.11	0.050	0.043	0.031
	B	0.08	0.14	0.16	0.19	< 0.001		
3D vector	A	0.33	0.50	0.61	0.73	< 0.001	−0.125	0.014
	B	0.27	0.43	0.44	0.53	< 0.001		
Roll	A	−0.06	−0.12	−0.05	−0.02	0.699	−0.038	0.143
	B	−0.02	−0.03	0.02	0.03	0.073		
Pitch	A	0.03	0.10	0.10	0.07	0.981	−0.048	0.041
	B	0.01	0.01	0.05	0.04	0.908		
Yaw	A	0.09	0.25	0.29	0.29	0.183	−0.125	0.032
	B	0.05	0.11	0.17	0.09	0.943		

X, superior-inferior direction; Y, right-left direction; Z, anterior-posterior direction; 3D, three-dimensional.

a The P value calculated from the Friedman ANOVA test.

b The unstandardized coefficient in the linear regression analysis.

c *The P value calculated from the linear regression analysis*.

The systematic errors (σ), random errors (σ), and estimated PTV margins (M) for the four 10-min sessions in both groups are listed in Table 3. We found that the estimated margins in three translational axes for Group A were all less than 1 mm within the first 10 min. As time went on, the margin required for the X and Y axes in Group A was more than 1 mm. On the other hand, the estimated margins in the three translational axes for Group B remained less than 1 mm for all four 10-min sessions.

**Table 3:** The systematic errors, random errors and estimated margins in the four 10-min sessions.

**Group**	**Session (min)**	**X (mm)**			**Y (mm)**			**Z (mm)**		
		**Σ**	**σ**	**M**	**Σ**	**σ**	**M**	**Σ**	**σ**	**M**
A	0–10	0.17	0.12	0.51	0.22	0.16	0.66	0.14	0.12	0.43
	11–20	0.29	0.10	0.80	0.38	0.12	1.03	0.17	0.09	0.49
	21–30	0.36	0.11	0.98	0.34	0.12	0.93	0.18	0.10	0.52
	31–40	0.47	0.22	1.33	0.48	0.15	1.31	0.20	0.14	0.60
B	0−10	0.09	0.13	0.32	0.16	0.13	0.49	0.15	0.12	0.46
	11–20	0.27	0.13	0.77	0.28	0.16	0.81	0.21	0.11	0.60
	21–30	0.24	0.11	0.68	0.21	0.18	0.65	0.19	0.12	0.56
	31–40	0.37	0.09	0.99	0.25	0.14	0.72	0.21	0.10	0.60

*X, superior-inferior direction; Y, right-left direction; Z, anterior-posterior direction; Σ, systematic errors; σ, random errors; M, estimated margins*.

**Figure 4:**
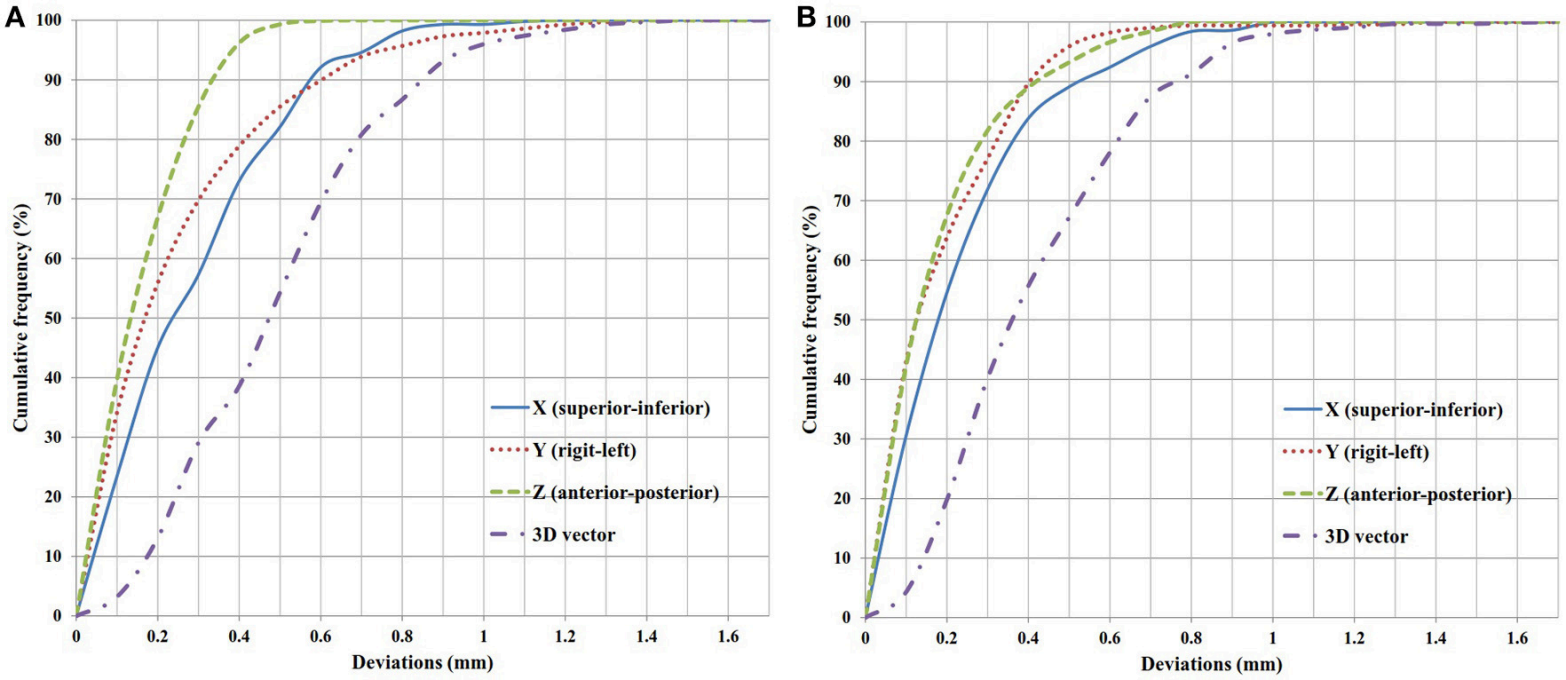
Cumulative frequency of translational deviations. **(A)** Group A. **(B)** Group B.

The cumulative frequency of errors with continuous increments of 0.1 mm of deviation in the translational axes are plotted in Figure 4. All of the deviations were calculated by the absolute values, regardless of the direction. To cover the 95% confidence interval for the deviations within 40 min, the margins of 0.77 mm in the X axis, 0.79 mm in the Y axis and 0.40 mm in the Z axis were needed in Group A, and 0.69 mm in the X axis, 0.50 mm in the Y axis, and 0.51 mm in the Z axis were needed in Group B. In clinical practice, if a margin of 1 mm was added to each axis, there would be 0.7% in the X axis and 2.1% in the Y axis exceeding 1 mm in Group A, and 0.6% in the Y axis exceeding 1 mm in Group B.

## Discussion

This is the first study to compare the intrafractional skull motions between different head supports in the frameless radiosurgery by CyberKnife. In the literature, one study reported by Li et al. (8) compared two immobilization systems, PinPoint system and Freedom system, for frameless radiosurgery by Trilogy (Varian Medical, Palo Alto, CA, USA) using optical surface imaging for motion monitoring. Another study reported by Tryggestad et al. (11) evaluated four thermoplastic mask-based immobilization systems for intracranial radiotherapy by Synergy-S (Elekta, Stockhom, Sweden) using cone-beam CT. However, there was no previous study which compared the different head supports in the frameless radiosurgery.

This study compared the different head supports during frameless radiosurgery by CyberKnife. Several studies reported intrafractional head motions in intensity-modulated radiation therapy (9, 12, 13). However, the intrafractional deviations reported by these studies were based on short treatment times in the conventionally fractionated radiation therapy. By repeat stereoscopic imaging with CyberKnife, Hoogeman et al. (14) proposed that systemic error increased nearly linearly with time to 0.5 mm within 15 min in the superior-inferior and left-right directions. As for the evaluation of long treatment times, one retrospective study reported that the skull motions would consistently increase over time in the frameless radiosurgery, especially in the superior-inferior direction (15). Another case series study also reported that the maximum displacement was in the longitudinal direction (16). Likewise, our study found that the progressive change in skull motion was significant not only in the superior-inferior direction in both groups but also in the anterior-posterior direction in Group B.

We found that skull motions in the superior-inferior direction, 3D vector, Pitch and Yaw in Group B were significantly smaller than those in Group A; conversely, the motions in the Z axis in Group B were significantly larger than those in Group A. It seemed that the head supports with cushions in Group B had better or similar results for immobilization in most axes, except in the anterior-posterior direction. This might be a result of the comfortable cushions added to the hard pillows so that patients in Group B could keep a stable position during the long treatment time, as the cushions might provide the opportunity to involuntarily move in the anterior-posterior direction. That's because the cushions settled and supported the neck posteriorly, superiorly, inferiorly, and bilaterally, which limited the involuntary movement toward the anterior direction.

The systemic errors would have more of an influence (Mptv = 2.5 Σ + 0.7σ) on calculating PTV margins than random errors. In this study, random errors (σ) in both groups had no obvious change over time, but systemic errors (Σ) showed obvious changes with time (Table 3). We found that PTV margins were small in the first 10-min session, and then increased with time, which might be due to the involuntary skull motions from the relaxation of head and neck muscles after the initial tension in the first 10-min session.

A previous study pointed out that, in the case of a thermoplastic mask system, a margin of about 1 mm was usually sufficient (11). Our data could validate this finding because there was only 0.7% in the X axis and 2.1% in the Y axis exceeding 1 mm in Group A, and 0.6% in the Y axis in Group B. To cover the 95% confidence intervals, the margins of three translational axes needed in both groups were all <1 mm.

Several involuntary movements outside the skull might lead to skull motions during treatment, such as swallowing, coughing, deep breathing, and so on. To diminish the influences of extreme values from these involuntary movements, we set every 2 min as a time point to analyze the data and averaged the data of three images around each time point; in addition, we also set four 10-min sessions to evaluate the changes in skull motions and estimate the margins.

There are several limitations of our study. First, the procedures of immobilization were not performed by the same therapist; therefore, the techniques for each therapist were not always the same, in spite of the standard operating procedure. Second, this study collected the image data within 40 min of each treatment. The results and conclusions of this study could therefore not stand for after 40 min. Third, as this was a retrospective study, there might be some selection bias in the patients enrolled in this study.

## Conclusion

Both head supports with thermoplastic masks could provide good immobilization during the frameless radiosurgery. To cover the 95% confidence intervals, the margins of three translational axes needed in both groups were all <1 mm. The patients in Group B had better or similar results for immobilization in most translational and rotational axes, except in the anterior-posterior direction.

## Ethics statement

This study was approved by the Chang Gung Medical Foundation Institutional Review Board (No. 201601207B0).

## Author contributions

CLK, SCL, YJH, FMF, and CCH were involved in the conception and design. CLK, SCL, JCW, KCL, TIL, KJJ, and CCH involved in the analysis and interpretation of the data. CLK, JCW, KCL, TIL, and KJJ drafted the paper. SCL, YJH, FMF, and CCH revised it critically for intellectual content. All authors gave their final approval of the version to be published.

### Conflict of interest statement

The authors declare that the research was conducted in the absence of any commercial or financial relationships that could be construed as a potential conflict of interest.

## References

[1] Adler JR Jr Chang SD Murphy MJ Doty J Geis P Hancock SL. The Cyberknife: a frameless robotic system for radiosurgery. Stereotact Funct Neurosurg. (1997) 69:124–8. 10.1159/000099863 9711744

[2] Murphy MJ. An automatic six-degree-of-freedom image registration algorithm for image-guided frameless stereotaxic radiosurgery. Med Phys. (1997) 24:857–66. 10.1118/1.598005 9198019

[3] Adler JR Jr Murphy MJ Chang SD Hancock SL. Image-guided robotic radiosurgery. Neurosurgery (1999) 44:1299–306, discussion 1306–7. 10371630

[4] Ramakrishna N Rosca F Friesen S Tezcanli E Zygmanszki P Hacker F. A clinical comparison of patient setup and intra-fraction motion using frame-based radiosurgery versus a frameless image-guided radiosurgery system for intracranial lesions. Radiother Oncol. (2010) 95:109–15. 10.1016/j.radonc.2009.12.030 20116123

[5] Masi L Casamassima F Polli C Menichelli C Bonucci I Cavedon C. Cone beam CT image guidance for intracranial stereotactic treatments: comparison with a frame guided set-up. Int J Radiat Oncol Biol Phys. (2008) 71:926–33. 10.1016/j.ijrobp.2008.03.006 18514784

[6] Guckenberger M Baier K Guenther I Richter A Wilbert J Sauer O. Reliability of the bony anatomy in image-guided stereotactic radiotherapy of brain metastases. Int J Radiat Oncol Biol Phys. (2007) 69:294–301. 10.1016/j.ijrobp.2007.05.030 17707284

[7] Baumert BG Egli P Studer S Dehing C Davis JB. Repositioning accuracy of fractionated stereotactic irradiation: assessment of isocentre alignment for different dental fixations by using sequential CT scanning. Radiother Oncol. (2005) 74:61–6. 10.1016/j.radonc.2004.08.002 15683671

[8] Li G Ballangrud A Chan M Ma R Beal K Yamada Y. Clinical experience with two frameless stereotactic radiosurgery (fSRS) systems using optical surface imaging for motion monitoring. J Appl Clin Med Phys. (2015) 16:149–62. 10.1120/jacmp.v16i4.5416 26219007 PMC4998054

[9] Linthout N Verellen D Tournel K Storme G. Six dimensional analysis with daily stereoscopic x-ray imaging of intrafraction patient motion in head and neck treatments using five points fixation masks. Med Phys. (2006) 33:504–13. 10.1118/1.2165417 16532958

[10] van Herk M Remeijer P Rasch C Lebesque JV. The probability of correct target dosage: dose-population histograms for deriving treatment margins in radiotherapy. Int J Radiat Oncol Biol Phys. (2000) 47:1121–35. 10.1016/s0360-3016(00)00518-6 10863086

[11] Tryggestad E Christian M Ford E Kut C Le Y Sanguineti G. Inter- and intrafraction patient positioning uncertainties for intracranial radiotherapy: a study of four frameless, thermoplastic mask-based immobilization strategies using daily cone-beam CT. Int J Radiat Oncol Biol Phys. (2011) 80:281–90. 10.1016/j.ijrobp.2010.06.022 20951506

[12] Suzuki M Nishimura Y Nakamatsu K Okumura M Hashiba H Koike R. Analysis of interfractional set-up errors and intrafractional organ motions during IMRT for head and neck tumors to define an appropriate planning target volume (PTV)- and planning organs at risk volume (PRV)-margins. Radiother Oncol. (2006) 78:283–90. 10.1016/j.radonc.2006.03.006 16564594

[13] Kim S Akpati HC Kielbasa JE Li JG Liu C Amdur RJ. Evaluation of intrafraction patient movement for CNS and head & neck IMR. Med Phys. (2004) 31:500–6. 10.1118/1.1644641 15070246

[14] Hoogeman MS Nuyttens JJ Levendag PC Heijmen BJ. Time dependence of intrafraction patient motion assessed by repeat stereoscopic imaging. Int J Radiat Oncol Biol Phys. (2008) 70:609–18. 10.1016/j.ijrobp.2007.08.066 17996389

[15] Wang CW Lin YC Tseng HM Xiao F Chen CM Cheng WL. Prolonged treatment time deteriorates positioning accuracy for stereotactic radiosurgery. PLoS ONE (2015) 10:e0123359. 10.1371/journal.pone.0123359 25894841 PMC4404334

[16] Bichay TJ Mayville A. The continuous assessment of cranial motion in thermoplastic masks during cyberknife radiosurgery for Trigeminal Neuralgia. Cureus (2016) 8:e607. 10.7759/cureus.607 27330875 PMC4905702

